# Availability of 24-h urine collection method on dietary phosphorus intake estimation

**DOI:** 10.3164/jcbn.16-50

**Published:** 2016-12-06

**Authors:** Masae Sakuma, Yuuka Morimoto, Yukie Suzuki, Akitsu Suzuki, Saaya Noda, Kanaho Nishino, Sakiko Ando, Makoto Ishikawa, Hidekazu Arai

**Affiliations:** 1 Laboratory of Clinical Nutrition and Management, Graduate School of Nutritional and Environmental Sciences, The University of Shizuoka, 52-1 Yada, Suruga-ku, Shizuoka 422-8526, Japan; 2 School of Nursing Sciences, The University of Shizuoka, 52-1 Yada, Suruga-ku, Shizuoka 422-8526, Japan

**Keywords:** dietary phosphorus, 24-h urine collection, urine phosphorus excretion, urine nitrogen excretion, loading test

## Abstract

Accurate assessment of dietary phosphorus intake is necessary to prevent hyperphosphatemia. The aim of this study was to evaluate the 24-h urine collection method for estimation of phosphate intake in healthy males. Two experiments, a 1-day and a 5-day loading test, were performed. After an overnight fast, subjects consumed test meals, 24-h urine collection was performed, and blood samples were obtained. In the 5-day loading test, a phosphorus supplement was orally administered on day 3. The association between the phosphorus content of test meals and urinary excretion, anthropometric indices, and blood biomarkers was analyzed to develop a more precise formula for estimating phosphorus intake. In the 1-day loading test, the standard deviation of predictive phosphorus intake, based on multiple linear regression analysis, was less than that for the phosphorus absorption rate. In the 5-day loading test, urinary phosphorus excretion was similar on days 2, 4 and 5, but was significantly higher on day 3 after phosphorus supplementation. Our results indicate that estimation of dietary phosphorus intake with the 24-h urine collection method, using the amount of phosphorus and urea nitrogen excretion, may increase the precision of short-term monitoring.

## Introduction

Elevated serum phosphorus levels are known to promote vascular calcification, arterial sclerosis and cardiovascular diseases,^(1–3)^ and have been associated with mortality in dialysis patients.^(4,5)^ Evidence suggests that hyperphosphatemia may induce cardiovascular events, even in individuals with normal renal function.^(6–8)^ Therefore, it is recommended that high dietary intake of phosphorus should be avoided to maintain serum phosphorus levels within the appropriate range. To prevent hyperphosphatemia, it is necessary to measure dietary phosphorus intake not only patients with renal dysfunction but also individuals with normal renal function.

Nutrient intake is usually assessed by weighed dietary records. However, this method may not enable accurate estimation of phosphorus intake, partly because of the lack of labeling requirements for phosphorus content on processed foods, which include phosphorus as an additive,^(9)^ and many processed foods are not listed in food composition tables. Furthermore, weighed dietary records do not reflect fluctuations in phosphorus content due to cooking method, production region and picking seasons.

Results from a previous study demonstrated that 24-h urine collection was superior for estimation of dietary phosphorus intake compared with weighed dietary records.^(10)^ However, in our previous study, the accuracy of the estimation of phosphorus intake was unknown because it had not compared the actual phosphorus intake. To improve the accuracy of estimating dietary phosphorus intake, further studies are required on the 24-h urine collection method. It is also necessary to determine whether 24-h urine collection reflects phosphorus intake over a single day or a few days. Therefore, in this study, healthy males underwent loading tests with meals of known phosphorus content, and factors affecting the urinary phosphorus excretion rate were evaluated.

## Materials and Methods

### Subjects and protocol

The study was performed after obtaining written informed consent from all subjects, and the protocol was approved by the Ethics Committee of the University of Shizuoka. The protocol conformed to the Helsinki Declaration.

### Single-day loading test

For this experiment, 15 healthy males without pre-existing conditions or medication use were enrolled. The clinical and biological characteristics of the subjects are shown in Table 1. The mean values ± SD for age and body mass index (BMI) were 22.9 ± 1.8 years and 22.2 ± 2.5 kg/m^2^, respectively. On the day prior to experiments, subjects were requested to refrain from heavy exercise and alcohol consumption, and to fast from 14:30 onwards. A pre-specified meal was provided at 18:30. After an overnight fast, subjects visited the laboratory at 07:15 and fasting venous blood samples were collected at 07:30. Subjects then consumed a test meal with known phosphorus content at 07:30, 12:30 and 17:30. The nutrient composition of the test meal was: 1,998 kcal/day, 14.5% protein, 18.6% fat, 66.9% carbohydrate, 1,200 mg/day phosphorus and 645 mg/day calcium. All subjects underwent 24-h urine collection from 07:30 to 07:30 the following morning. Blood samples were collected at 07:30 the following morning.

### Five-day loading test

This experiment included 8 healthy males without pre-existing conditions or medication use. The clinical and biological characteristics of the subjects are shown in Table 2. The mean values ± SD for age and BMI were 22.8 ± 1.6 years and 21.3 ± 2.2 kg/m^2^, respectively.

**Fig. 1:**
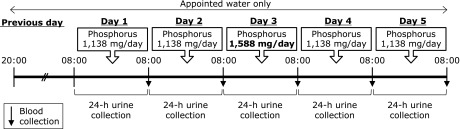
Schema of 5-day loading test.

An outline of the study protocol is shown in Fig. 1. This experiment was conducted over 6 consecutive days. On the day prior to the experimental period, subjects were asked to avoid heavy exercise and alcohol consumption, and to fast from food and beverages other than water after 20:00. After the overnight fast, subjects visited the laboratory at 08:00, and consumed a test meal at 08:30, 12:30 and 18:30. Nutrient composition of the test meals are shown in Table 3. Urine samples were collected over 24-h from 08:00 to 08:00 the following morning, and blood samples were obtained at 08:15 the following morning. For the next 5 days, subjects consumed three test meals, and underwent 24-h urine collection and blood sampling on each day. On day 3, a phosphorus supplement (150 mg) was orally administered as a solution of neutral sodium phosphate (mixture of Na_2_HPO_4_ and NaH_2_PO_4_) with each meal (total 450 mg/day).

### Anthropometric and blood analysis

Height, body weight, body fat mass and body fat percentage were calculated using a bioelectrical impedance analysis method (TANITA TBF-215; TANITA Corporation, Tokyo, Japan). BMI and body surface area (BSA) were calculated using the following formulae:

BMI = body weight (kg)/body height (m)^2^

BSA = body weight^0.425^ (kg) × body height^0.725^ (cm) × 0.007184

Blood samples were dispensed into vacuum tubes and immediately centrifuged (4°C, 1,500 *g*, 10 min). Sera and plasma were separated, and samples were stored at −30°C until analysis of triglycerides (TG), low-density lipoprotein cholesterol (LDL-Cho), high-density lipoprotein cholesterol (HDL-Cho), hemoglobin A_1_c (HbA_1_c), albumin, aspartate aminotransferase (AST), alanine aminotransferase (ALT), γ-glutamyl transpeptidase (γ-GTP), blood urea nitrogen (BUN), serum creatinine (S-Cre), serum sodium (S-Na), potassium (K), chloride (Cl), serum calcium (S-Ca), serum phosphorus (S-Pi), intact parathyroid hormone (iPTH), and fibroblast growth factor 23 (FGF23) by a commercial laboratory (SRL Inc., Tokyo, Japan).

### 24-h urine collection method

Urine samples were collected over a 24-h interval. Subjects were instructed to discard the first morning void and to collect all urine over the next 24-h, including the first void on the following morning. After recording the total volume, urine samples were dispensed into vessels for storage at 4°C or –30°C until analysis. Urine nitrogen (U-UN), urine creatinine (U-Cre), urine sodium (U-Na), urine calcium (U-Ca) and urine inorganic phosphorus (U-Pi) levels were measured by a commercial laboratory (SRL Inc.). Creatinine clearance (CCr) and the phosphorus absorption rate were calculated by the following formulae:

CCr (ml/min) = [U-Cre (mg/dl) × urine volume (ml/min)/S-Cre (mg/dl)] × 1.73/BSA (m^2^)

Phosphorus absorption rate (%) = U-Pi (mg/day)/content of phosphorus in test meal (TM-P) (mg) × 100

### Statistical analysis

Data are expressed as the means ± SD. *P* values<0.05 were considered to denote statistical significance. The Shapiro-Wilk test was used to determine normality. The phosphorus absorption rate of subjects was calculated and defined as 75%, and estimated as UC-P75. Pearson’s product-moment correlation coefficient and Spearman’s rank-correlation coefficient were used to evaluate the relationship between UC-P75 and TM-P. Factors that influenced UC-P75 – TM-P were evaluated by multiple linear regression analysis. Calculation of predicted phosphorus intake (Pre-P) was based on the results of multiple linear regression analysis. Differences between UC-P75 – TM-P and Pre-P – TM-P were assessed using the Student’s *t* test for paired comparisons. Transitional changes in urine and serum indices on phosphorus metabolism were calculated using a repeated measure one-way analysis of variance (ANOVA) or Friedman’s test with Tukey’s post hoc test. All statistical analyses were performed using SPSS ver. 19.0 software (SPSS Inc., Chicago, IL).

## Results

### Single-day loading test

Mean daily excretion of U-Pi, U-UN, U-Cre, U-Na and U-Ca was 900.0 ± 119.9 mg, 9.4 ± 1.9 g, 1,648.0 ± 278.1 mg, 2,986.7 ± 595.1 mg and 158.7 ± 45.2 mg, respectively. The estimated mean phosphorus absorption rate was 74.9 ± 10.0%. The phosphorus absorption rate was defined as 75%, and UC-P75 was calculated with the following formula:

UC-P75 (mg/day) = U-Pi (mg/day) × 100/75

The difference between UC-P75 and TM-P (UC-P75 – TM-P) was –1 ± 160 mg/day, with very high inter-individual variability. To evaluate the association of UC-P75 – TM-P with anthropometric indices, and blood and urine biomarkers, correlation analyses were performed. UC-P75 – TM-P was positively correlated with body weight, body fat mass, BSA, U-UN and U-Cre (Table 4). Multiple linear regression analysis demonstrated that U-UN was a significant and independent factor (Table 5), and the following equations were generated:

UC-P75 – TM-P (mg/day) = U-UN (g/day) × 63.668 – 596.671

Pre-P (mg/day) = 100/75 × U-Pi (mg/day) – [U-UN (g/day) × 63.668 – 596.671]

**Fig. 2:**
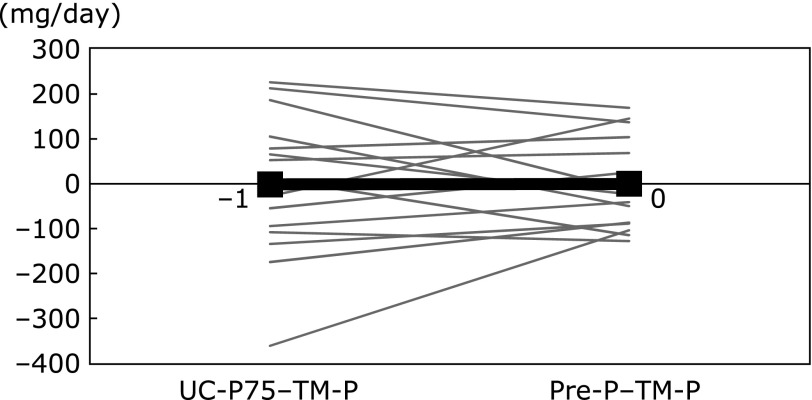
Difference in phosphorus intake using two methods. TM-P, phosphorus intake from test meal; UC-P75, phosphorus intake estimated from 24-h urine collection; Pre-P, predicted phosphorus intake. Black and bold line, mean value.

The differences between TM-P and UC-P75 or Pre-P were −1 ± 160 mg/day or 0 ± 102 mg/day, respectively. There were no differences in the mean values, but Pre-P had lower SD than UC-P75 (Fig. 2).

### Five-day loading test

**Fig. 3:**
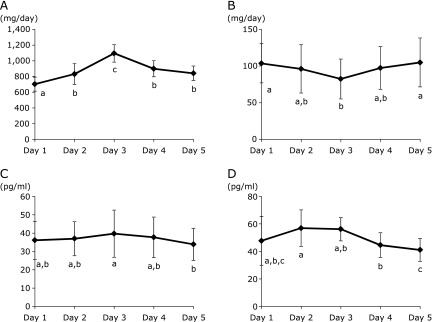
Transitional changes in urine and serum indices of phosphorus metabolism. (A) urine phosphorus excretion, (B) urine calcium excretion, (C) serum iPTH levels, (D) serum FGF23 levels. iPTH, intact parathyroid hormone; FGF23, fibroblast growth factor 23. Values with different alphabet are significantly different at *p*<0.05 by repeated measure one-way analysis of variance (ANOVA) or Friedman’s test with Tukey’s post hoc test.

On days 1, 2, 3, 4 and 5, daily U-Pi was 703.8 ± 88.8 mg, 832.5 ± 133.5 mg, 1,096.3 ± 111.7 mg, 900.0 ± 102.4 mg and 842.5 ± 92.2 mg, respectively (Fig. 3A). U-Pi was significantly higher on day 3 than the other days, with values on days 2, 4 and 5 similar to each other. U-Ca excretion was significantly lower on day 3 than days 1 and 5 (Fig. 3B).

S-Pi and S-Ca were not altered during the study period (data not shown). Serum iPTH levels were slightly elevated on day 3 after phosphorus supplementation, and were significantly higher on day 3 compared with day 5 (Fig. 3C). Serum FGF23 levels were not altered by phosphorus intake (Fig. 3D).

## Discussion

The difference between UC-P75 (calculated based on an absorption rate of 75%) and TM-P (phosphorus content of test meal) (UC-P75 – TM-P) revealed a high degree of inter-individual variability (–361~226 mg/day). This may be explained by differences in the ability to liberate and absorb phosphorus from foods, and body size or composition.

The first possible factor was the ability to liberate phosphorus from foods. Phosphorus is classified as inorganic and organic. Inorganic phosphorus exists as part of a phosphate compound, and it is easily liberated and absorbed.^(11,12)^ On the other hand, organic phosphorus, which is found in substances such as animal protein, is esterified and binds to amino acid side chains.^(13)^ Phosphate esters are hydrolyzed to inorganic phosphorus and alcohol by alkaline phosphatase (ALP), and inorganic phosphorus is absorbed from the intestine. A previous study reported that ingestion of different esterified phosphate content for 5 weeks increased ALP activity in the upper part of the small intestine of growing rats in an esterified phosphate content-dependent manner.^(14)^ In contrast, another study found that increasing phosphorus intake led to reduced ALP activity.^(15)^ In this study, the dietary habits of subjects may have contributed to individual differences in digestive enzyme activity.

Another potential factor related to inter-individual variability is differences in absorption of dietary phosphorus from the intestine, which occurs via active and/or passive transport processes. Active transport is mediated by the type II sodium-dependent phosphate cotransporter NaPi-2b. A low phosphorus diet has been reported to increase NaPi-2b mRNA expression in mouse small intestine.^(16)^ Thus, the habitual phosphorus intake of the subjects may have led to differences in their ability to absorb intestinal phosphorus.

A third factor was physical constitution, with UC-P75 – TM-P being positively correlated with body weight, body fat mass and BSA. It has been suggested that adjustments should be made for individual physical constitution when estimating phosphorus intake using the 24-h urine collection method.

Multiple linear regression analysis demonstrated that inter-individual variability in UC-P75 – TM-P was positively associated with U-UN. Other studies have identified a strong positive correlation between U-Ca and U-UN (from protein intake) due to an increase in intestinal calcium absorption.^(17,18)^ The absorption rate of both phosphorus and calcium may be influenced by protein intake. U-UN has been suggested to reflect protein intake.^(17)^ Because organic phosphorus tends to bind to protein, U-UN may correspond to inter-individual differences in the digestion and absorption of organic phosphorus.

There were no differences between TM-P and UC-P75 or Pre-P, but SD was lower for Pre-P than UC-P75. Thus, Pre-P was superior to UC-P75 for estimation, with a coefficient of variance of 8.5. Because the coefficient of variation of protein intake according to Maroni’s formula was 18.7 (data not shown), Pre-P was considered to provide a reasonable estimation of phosphorus intake.

In the 5-day loading test, U-Pi excretion after the administration of phosphorus supplements was significantly higher on day 3 than day 2. It has been suggested that iPTH secretion increases in parallel with phosphorus intake, and urinary phosphorus excretion was elevated on day 3. U-Pi on days 4 and 5 was significantly lower than on day 3, and was similar to day 2. This suggests that dietary phosphorus is completely excreted via the urine in approximately 1 day, and estimation of phosphorus intake using the 24-h urine collection method is appropriate for short-term evaluation of phosphorus intake.

Limitations of this study are that only a small number of healthy young male subjects were investigated. Thus, the results may not be applicable to females, elderly individuals, and those with renal failure. Further research is planned across a wider spectrum of the population, including females, different age groups and patients with renal dysfunction. Also, the studies of various phosphorus intakes are needed.

The present findings indicate that estimation of daily dietary phosphorus intake with the 24-h urine collection method, using the amount of U-Pi and U-UN excretion, was accurate, and the 24-h urine collection method is most suitable for short-term estimation of phosphorus intake.

## Figures and Tables

**Table 1:** Characteristics of the subjects in a loading test of a single day

Characteristic		mean ± SD
Age	(year)	22.9 ± 1.8
Body weight	(kg)	63.7 ± 9.5
Body fat percentage	(%)	18.1 ± 3.6
Body mass index	(kg/m^2^)	22.2 ± 2.5
Triglyceride	(mg/dl)	71.8 ± 22.7
LDL-cholesterol	(mg/dl)	82.0 ± 15.1
HDL-cholesterol	(mg/dl)	60.8 ± 11.4
HbA_1_c (NGSP)	(%)	5.2 ± 0.3
Albumin	(g/dl)	4.8 ± 0.3
AST	(U/L)	16.5 ± 3.5
ALT	(U/L)	12.8 ± 3.1
γ-GTP	(U/L)	16.5 ± 4.2
Blood urea nitrogen	(mg/dl)	11.4 ± 2.5
Creatinine	(mg/dl)	0.9 ± 0.1
Ccr	(ml/min)	129.1 ± 8.8
Na	(mEq/L)	140.3 ± 1.2
K	(mEq/L)	4.2 ± 0.1
Ca	(mg/dl)	9.6 ± 0.4
Pi	(mg/dl)	4.0 ± 0.4
intact PTH	(pg/ml)	39.7 ± 11.2

Values are mean ± SD. AST, aspartate aminotransferase; ALT, alanine aminotransferase; γ-GTP, γ-glutamyl transpeptidase; Ccr, creatinine clearance; Na, sodium; K, potassium; Ca, calcium; Pi, phosphorus; iPTH, intact PTH.

**Table 2:** Characteristics of the subjects in a loading test of 5 days

Characteristic		mean ± SD
Age	(year)	22.8 ± 1.6
Body weight	(kg)	64.2 ± 8.5
Body fat percentage	(%)	17.6 ± 4.3
Body mass index	(kg/m^2^)	21.3 ± 2.2
Triglyceride	(mg/dl)	94.1 ± 38.3
LDL-cholesterol	(mg/dl)	80.0 ± 20.5
HDL-cholesterol	(mg/dl)	52.3 ± 11.8
HbA_1_c (NGSP)	(%)	5.2 ± 0.3
Albumin	(g/dl)	4.7 ± 0.3
AST	(U/L)	19.8 ± 3.4
ALT	(U/L)	18.5 ± 4.1
γ-GTP	(U/L)	21.6 ± 6.8
Blood urea nitrogen	(mg/dl)	11.2 ± 1.9
Creatinine	(mg/dl)	0.9 ± 0.1
Ccr	(ml/min)	121.7 ± 10.1
Na	(mEq/L)	141.3 ± 0.7
K	(mEq/L)	4.4 ± 0.5
Ca	(mg/dl)	9.6 ± 0.3
Pi	(mg/dl)	3.9 ± 0.2
intact PTH	(pg/ml)	36.1 ± 10.3

Values are mean ± SD. AST, aspartate aminotransferase; ALT, alanine aminotransferase; γ-GTP, γ-glutamyl transpeptidase; Ccr, creatinine clearance; ALP, alkaline phosphatase; Na, sodium; K, potassium; Ca, calcium; Pi, phosphorus; iPTH, intact PTH.

**Table 3:** Composition of the test meals

	Energy (kcal)	Protein (g)	Fat (g)	Carbohydrate (g)	Phosphorus (mg)	Calcium (mg)
Breakfast	668	28.0	18.2	104.9	473	348
Lunch	753	19.6	16.9	130.2	240	92
Dinner	716	22.6	14.4	124.1	425	180
Total	2,137	70.2	49.2	359.2	1,138	620
%Energy		13.1	20.7	67.2		

**Table 4:** Pearson’s correction coefficients of difference between estimated phosphorus and dietary phosphorus with metabolic variables

	UC-P75 – TM-P
Body weight	0.54*
Body fat percentage	0.47
Body fat	0.52*
Fat free mass	0.49
Body surface area	0.55*
Ccr	0.44
24-h urinary UN excretion	0.77**
24-h urinary Cre excretion	0.53*
24-h urinary Na excretion	–0.02
24-h urinary Ca excretion	0.39
Serum Pi	–0.17
Serum Ca^#^	0.19
Serum intact PTH	–0.12
Serum Cre	0.15
Serum total protein	0.27
Serum albumin	0.28

^#^Spearman’s correction coefficient. *p<0.05, **p<0.01. UC-P75, phosphorus intake estimated from 24-h urine as phosphorus absorption rate is 75%; TM-P, posphorus intake from test meal; Ccr, creatinine clearance; Pi, phosphorus; UN, urine nitrogen; Cre, creatinine; Na, sodium; Ca, calcium.

**Table 5:** Association between difference of phosphorus intake with metabolic variables

		Difference of phosphorus intake			
	non-standardization coefficient	95%CI		β	*p* value
		Lower	Upper		
(Constant)	–596.671	–896.661	–296.680		0.001
24-h urinary UN	63.668	32.223	95.113	0.772	0.001

Data were used multiple linear regression analysis. UN, urine nitrogen. Adjusted R^2^ variation; 0.564.

## Abbreviations

Alb: albumin
ALT: alanine aminotransferase
AST: aspartate aminotransferase
BMI: body mass index
BSA: body surface area
BUN: blood urea nitrogen
CCr: creatinine clearance
Cl: chloride
FGF23: fibroblast growth factor 23
γ-GTP: γ-glutamyl transpeptidase
HbA_1_c: hemoglobin A_1_c
HDL-Cho: high-density lipoprotein cholesterol
iPTH: intact parathormone
K: potassium
LDL-cho: low-density lipoprotein cholesterol
Pre-P: predictive phosphorus intake
S-Ca: serum calcium
S-Cre: serum creatinine
S-Na: serum sodium
S-Pi: serum phosphorus
TG: triglyceride
TM-P: phosphorus in test meal
U-Ca: urine calcium
U-Cre: urine creatinine
U-Na: urine sodium
U-Pi: urine inorganic phosphorus
U-UN: urine nitrogen
